# Analysis of integrons and associated gene cassettes in clinical isolates of multidrug resistant *Pseudomonas aeruginosa* from Southwest Nigeria

**DOI:** 10.1186/1476-0711-12-29

**Published:** 2013-10-21

**Authors:** Bamidele T Odumosu, Bolanle A Adeniyi, Ram Chandra

**Affiliations:** 1 Department of Pharmaceutical Microbiology, University of Ibadan, Ibadan, Nigeria; 2 Environmental Microbiology Section, CSIR-Indian Institute of Toxicology Research, Lucknow, India

**Keywords:** *Pseudomonas aeruginosa*, Antibiotic resistance, Plasmids, Integrons, Gene cassettes

## Abstract

**Background:**

Multidrug resistant *Pseudomonas aeruginosa* harbours integrons and other mobile genetic elements such as plasmids and transposons, which easily disseminate antibiotic resistance genes among clinical strains of *P. aeruginosa.*

**Methodology:**

Plasmid extraction of 54 clinical isolates of *P. aeruginosa* was carried out by alkaline lysis method; and plasmid size estimation was done by using *E. coli* V517 standard plasmid marker. Fifty-four clinical strains of *P. aeruginosa* were isolated from 5 hospitals in 3 Southwestern states of Nigeria between March and September 2010. Plasmid extraction of isolates was carried out by alkaline lysis method; and plasmid size estimation was done by using *E. coli* V517 standard plasmid marker. PCR amplification for the 3 classes of resistance integrons, and gene cassette characterization were carried out using specific primers and by sequencing of PCR products. Conjugal mating of the integron positive *P. aeruginosa* strains with *E. coli* DH5α was performed to demonstrate transferability of integrons and gene cassettes.

**Result:**

Agarose gel electrophoresis of plasmid DNA revealed that all the 54 *P. aeruginosa* harboured 1–4 plasmids with sizes ranging from 2.2 – >58 kb. Class 1 integron was identified in 31 (57%) strains; but none of them carried class 2 and class 3 integrons. High prevalence of *aadA* gene conferring resistance to streptomycin/spectinomycin was detected in the strains positive for class 1 integron. Sequencing of the 1.6 kb and 1.2 kb amplified band of gene cassettes revealed the presence of *aadA6-orfD* and *aadA13* respectively.

**Conclusion:**

This study demonstrates the presence of plasmids and integrons harbouring resistance gene cassettes, which may collectively constitute an efficient system for dissemination of resistance genes in *P. aeruginosa*. Disturbingly, the rapid and unabated spread of class 1 integron-associated multidrug resistant *P. aeruginosa* in Southwest Nigeria may greatly hamper successful treatment of infections caused by such strains. This necessitates the establishment of functional antimicrobial resistance surveillance programmes in Nigeria.

## Introduction

The worldwide threat of nosocomial multidrug resistant *Pseudomonas aeruginosa* is a growing concern among hospitalized patients. Infections caused by *P. aeruginosa* are severe and mostly associated with high mortality and morbidity rates. *P. aeruginosa* frequently develops resistance during therapy; hence, it becomes difficult to treat [1]. *P. aeruginosa* is naturally resistant to many structurally unrelated antibiotics because of its dominant array of chromosomal and plasmid-mediated antibiotic resistance factors [1]; as well as its dynamic propensity to acquire newer resistance genes from bacteria from other genera such as *Acinetobacter baumannii, Klebsiella pneumoniae* and *Salmonella* spp [2].

Mobile genetic elements such as plasmids, transposons and integrons are means of acquiring resistance mechanisms contributing to *P. aeruginosa* multidrug resistance [2]. These mobile genetic elements play an important role in the dissemination of resistance genes among bacteria. Several studies have related antimicrobial resistance of *P. aeruginosa* to the presence of one or more of these genetic elements [3-6]. Integrons are genetic elements that capture and integrate gene cassettes by site-specific recombination and convert them to functional genes. The essential components of an integron include the integrase gene (*intI*), the gene for the adjacent recombination site (*attI*) and the promoter (*Pc*), which promotes the expression of any suitably integrated gene [7]. Genes carried by integrons usually encodes multiple resistance mechanisms such as resistance to beta-lactams, aminoglycosides and other antimicrobial agents [8,9].

There are three main well characterized classes of antibiotic resistance integrons designated class 1, 2 and 3 [10,11]. The class 1 integrons remain the most common integrons found in members of the family Enterobacteriaceae such as *Enterobacter* spp, *K. pneumoniae, E. coli* and *Proteus* spp., as well as other clinically important Gram-negative bacteria such as *P. aeruginosa* and *Acinetobacter baumannii *[6,12]. Detection of class 2 and class 3 integrons among these nosocomial pathogens is not widely reported [12]. Though, a recent study from Southern China found class 2 integron carrying Tn*7*-like *dfrA1-sat1-aadA1* conferring resistance to trimethoprim, streptothricin and streptomycin/spectinomycin respectively in clinical strains of *P. aeruginosa *[13].

In Nigeria, bacterial resistance to cheap and readily available antimicrobials has necessitated the wide use of broad-spectrum antibiotics [14]. Consequently, multidrug-resistant *P. aeruginosa,* as well as other resistant nosocomial pathogens have been associated with serious infections due to indiscriminate use of these broad-spectrum antibiotics [15]. Integrons, plasmids and other mobile genetic elements have been implicated in the evolution and dissemination of multidrug resistant bacteria [11,12]. However, reports of multidrug resistance in *P. aeruginosa* associated with mobile genetic elements are scanty in our region. The aim of this study was to investigate integron-associated resistance and characterize the respective gene cassettes among multidrug resistant *P. aeruginosa* from Nigeria.

## Materials and method

### Bacterial isolates

Fifty-four non-duplicate multidrug resistant *P. aeruginosa* strains isolated during a seven-month period (March - September 2010) from University College Hospital Ibadan (*n = 20*), Catholic Hospital Oluyoro Ibadan (*n = 12*), Catholic Hospital Eleta Ibadan (*n = 7*), Federal Medical Centre Akure (*n = 5*), Federal Medical Centre Abeokuta (n = 10) were included in this study. The isolates were obtained from different clinical samples (wound swab, urine, pus, ear swab, blood and vagina swab) and were verified using standard biochemical methods as described previously [16]. All isolates were collected under approved ethical standards.

### Antimicrobial susceptibility testing

Antimicrobial susceptibility was determined by the disk diffusion method on Mueller-Hinton agar (Oxoid UK), according to the Clinical and Laboratory Standards Institute guidelines [17]. Antimicrobial susceptibility testing was performed for 13 antimicrobial agents: ceftriaxone (CRO, 30 μg), ceftazidime (CAZ, 30 μg), cefotaxime (CTX, 30 μg), carbenicillin (CAR, 100 μg), piperacillin (PRL, 100 μg), levofloxacin (LEV, 5 μg), ciprofloxacin (CIP, 5 μg), gentamicin (GEN, 10 μg), amikacin (AMK, 30 μg), streptomycin (STR, 10 μg), tetracycline (TET, 30 μg), imipenem (IPM, 10 μg) and ticarcillin/clavulanic acid (75/10 μg). *P. aeruginosa* ATCC 27853 and *E. coli* ATCC 25922 were used for quality control of the susceptibility testing.

### Plasmid and genomic DNA extraction

Plasmid extraction was carried out by alkaline lysis method [18], with *E.coli* V517 used for plasmid size estimation as described previously [19]. Plasmid DNA bands were visualized and photographed by using Gel Documentation system with UV transillumination. Genomic DNA extraction was carried out as described previously [20] with modifications. Briefly, the *P. aeruginosa* isolates were inoculated into 2 ml of Trypticase Soy broth (Difco, Detroit, MI) and incubated overnight at 37°C. The bacterial cells were harvested by centrifugation at 8,000 × g for 5 min and the supernatant was completely removed using sterile Pasteur pipette. The pellet was resuspended in 500 μl of Tris EDTA (TE) buffer. The cells were lysed by boiling for 10 min in a water bath, cooled on ice, and centrifuged at 14,000 × g for 5 min to remove any cell debris before it was stored at -20°C. Aliquots of 2 μl of the template DNA were used for polymerase chain reaction (PCR).

### Integron detection

Class 1, 2 and 3 integrons were initially detected by PCR using degenerate primer set hep35 and hep36 (Table 1), which hybridizes to conserve regions of integron-encoded integrase genes *intI1, intI2*, and *intI3*[21]. The class of integron was determined by analyzing integrase PCR products through enzyme digestion using *Hinf*I as described previously [22]. Specific primers for class 2 and 3 integrons were also used in separate PCR assays [23] [Table 1].

**Table 1:** Primers used in this study

**Name**	**Sequence (5′-3′)**	**Target region**	**Expected size (bp)**	**Reference**
hep35 *	TGCGGGTYAARGATBTKGATTT	*Int-1,2,3*	491	[21]
hep36 *	CARCACATGCGTRTARAT	*Int-1,2,3*		
**hep58**	TCATGGCTTGTTATGACTGT	Cassette arrays in class 1 integrons	Variable	[21]
**hep59**	GTAGGGCTTATTATGCACGC	Cassette arrays in class 1 integrons		
**intI2L**	CACGGATATGCGACAAAAAGGT	Class 2 integron	789	[23]
**intI2R**	GTAGCAAACGAGTGACGAAATG	Class 2 integron		
**intI3L**	GCCTCCGGCAGCGACTTTCAG	Class 3 integron	980	[23]
**intI3R**	ACGGATCTGCCAAACCTGACT	Class 3 integron		

*For hep35 and hep36: B = C or G or T, K = G or T, R = A or G and Y = C or T.

### Characterization of class 1 integron cassette arrays and DNA sequencing

Class 1 integron cassette regions were amplified using hep58 and hep59 primers as described previously [21] using the following modified conditions; initial denaturation of 94°C for 2 min, 35 cycles of 94°C for 30 s, 55°C for 45 s, extension at 72°C for 45 s and final extension at 72°C for 7 min. Amplified gene cassette of the same fragment sizes, were further sequenced to determine the cassette array present. The amplicons were purified and sequenced at the sequencing facility of Chromous Biotech, Bangalore, India.

### Sequence analysis and nucleotide sequence accession number

Homology searches were performed with BLAST (http://www.ncbi.nlm.nih.gov). Sequences were assembled with BioEdit, and multiple sequences were aligned with Clustal W (http://www.ebi.ac.uk/). The sequences obtained from the gene cassette analysis have been deposited in GenBank/EMBL/DDBJ under accession numbers JX195555 for *aadA6-ofrD* of *P. aeruginosa* strain ODM-24 and JX195556 for *aadA13* of strain ODM-08.

### Conjugation assay

Conjugation was carried out using previously described protocol [24] with modification. Briefly, donor *P. aeruginosa* and recipient nalidixic acid-resistant *E. coli* DH5α cells were grown to logarithmic phase in Luria broth (LB), equal volume (500 μl) of each strains were mixed thoroughly in a tube and centrifuged at 5000 rpm for 3 min. The pellets were inoculated on Mueller Hinton agar plates containing no antibiotics and incubated at 37°C for 24 h. Transconjugants were selected on nutrient agar plates containing 30 μg nalidixic acid plus one of the antibiotics to which the donor strain was resistant as described previously [24].

## Results

### Susceptibility testing

Susceptibility testing of the 31 integron positive *P. aeruginosa* isolates showed that all the isolates were resistant to ≥ 5 of the 13 antimicrobial agents tested in this study. Resistance was mostly observed for tetracycline (100%), amoxicillin/clavulanate (100%), followed by streptomycin (90.3%), ceftriaxone (87.1%), kanamycin (83.8%), carbenicillin (80.6%), cefotaxime (77.4%) and to a lesser degree amikacin (25.5%), ceftazidime (22.5%), and imipenem (9.6%) (Table 2).

**Table 2:** **Antimicrobial Susceptibility of 31 class 1 integron positive *P. aeruginosa *** **isolates from Southwest Nigeria**

**Isolate I.D**	**Site of isolation**	**CTX**	**CAZ**	**CRO**	**CAR**	**PRL**	**AMK**	**GEN**	**STR**	**TIM**	**CIP**	**LEV**	**IPM**	**TET**	**Conjugation**
**ODM-01**	Urine	R	S	R	R	S	S	S	R	R	S	S	S	R	Positive
**ODM-03**	Urine	S	S	R	R	S	S	S	S	R	S	R	S	R	Negative
**ODM-04**	wound	S	S	S	R	S	R	S	S	R	R	R	S	R	Negative
**ODM-05**	Pus	S	S	R	S	S	S	S	S	R	S	R	S	R	Negative
**ODM-08**	Wound	R	S	R	R	S	S	R	R	R	R	S	S	R	Positive
**ODM-12**	Wound	R	R	R	R	R	R	R	R	R	R	R	R	R	Negative
**ODM-13**	Urine	R	S	R	R	S	S	S	R	R	S	S	S	R	Negative
**ODM-17**	Urine	R	S	R	R	R	S	S	R	R	S	S	S	R	Positive
**ODM-19**	Wound swab	S	S	S	S	S	S	R	R	S	S	S	S	R	Negative
**ODM-20**	Pus	R	S	R	R	R	S	S	R	R	R	R	S	R	Negative
**ODM-22**	Urine	S	S	S	S	S	S	S	R	S	S	R	S	R	Negative
**ODM-23**	Wound swab	R	S	R	R	S	S	S	R	R	R	R	S	R	Negative
**ODM-24**	Urine	R	S	R	R	R	S	R	R	R	R	R	S	R	Negative
**ODM-25**	Urine	R	R	R	R	R	S	R	R	R	R	R	S	R	Positive
**ODM-27**	Throat swab	R	S	R	R	R	S	S	R	R	R	R	S	R	Negative
**ODM-28**	Ear swab	R	R	R	R	S	R	R	R	R	S	S	S	R	Positive
**ODM-32**	Wound biopsy	R	R	R	S	S	R	R	R	R	S	S	S	R	Positive
**ODM-34**	Vaginal swab	S	S	R	R	R	R	S	R	R	R	R	S	R	Negative
**ODM-35**	Urine	R	R	R	R	R	S	S	R	R	S	R	S	R	Negative
**ODM-36**	Pus	R	S	R	R	S	S	S	R	R	R	R	S	R	Negative
**ODM-38**	Urine	R	S	R	R	R	R	R	R	R	R	R	S	R	Positive
**ODM-40**	Vaginal swab	R	S	R	R	R	R	R	R	R	R	R	S	R	Positive
**ODM-41**	Umbilical swab	R	S	R	S	S	S	R	R	R	S	S	R	R	Negative
**ODM-42**	Pus	R	R	R	R	R	S	S	R	R	S	R	S	R	Positive
**ODM-43**	pus	R	S	R	R	R	S	R	R	R	S	S	R	R	Negative
**ODM-45**	Wound swab	R	S	R	R	R	R	R	R	R	S	S	S	R	Negative
**ODM-46**	Urine	R	S	R	R	R	S	R	R	R	R	S	S	R	Positive
**ODM-48**	Urine	R	S	R	R	R	S	R	R	R	R	R	S	R	Negative
**ODM-49**	Urine	R	S	R	R	R	S	R	R	R	R	S	S	R	Negative
**ODM-52**	Urine	R	R	R	R	R	S	S	R	R	R	S	S	R	Negative
**ODM-54**	Wound swab	S	S	S	S	S	S	R	R	R	S	S	S	R	Negative

### Plasmid profile

Agarose gel electrophoresis of the plasmid DNA revealed that all the 54 *P. aeruginosa* strains harboured 1–4 plasmids with sizes ranging from 2.2 – >58 kb as compared with the corresponding *E. coli* V517 standard marker (Table 3). Eleven distinct plasmid profiles labelled A-K were identified. Thirty-seven (69%) *P. aeruginosa* strains carried a single large plasmid (58 kb or >58 kb) while 17 (32%) isolates harboured 2 – 4 variety of plasmids that differed in sizes (Table 3).

**Table 3:** **Plasmid Profiles of 54 *P. aeruginosa *isolates from Southwest Nigeria**

**Plasmid pattern**	**Plasmid sizes (Kb)**	**No of isolates**
A	58	31
B	>58	6
C	58, >58	9
D	2.2, >58	1
E	2.7, >58	1
F	3.2, >58	1
G	2.2, 3.2, 58, >58	1
H	2.4, 3.2, 58, >58	1
I	2.7, 3.2, 58	1
J	2.2, 17, 58	1
K	17, 58, > 58	1

### *Int* genes

**Figure 1:**
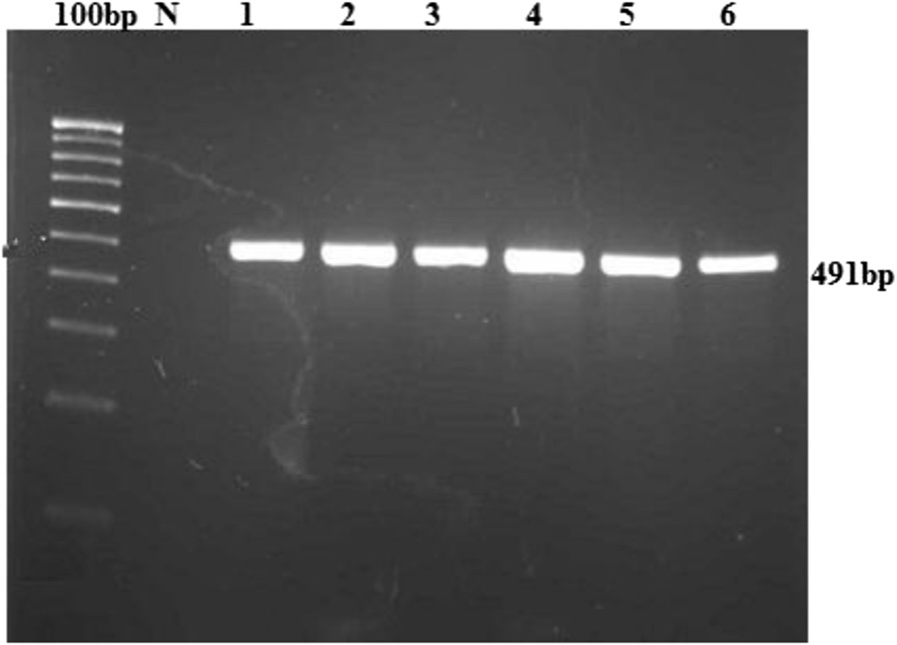
**Agarose gel electrophoresis of *Int *** **gene universal PCR product (1.8% agarose in 5x TAE) Lane N, negative control, Lane 1 – 7 integron positive strains of *P. aeruginosa*.**

PCR assay with primers hep35 and hep36 revealed that 31 (57%) of the 54 *P. aeruginosa* strains were integrase positive (Figure 1). Analysis of the integrase PCR product by restriction fragment length polymorphism (RFLP) by *Hin*fI also yielded a single fragment of 491 bp among the 31 strains, confirming class 1integron as described previously [21,22]. None of the isolates were positive for class 2 and 3 integrons specific PCR assays.

### Gene cassette array

**Figure 2:**
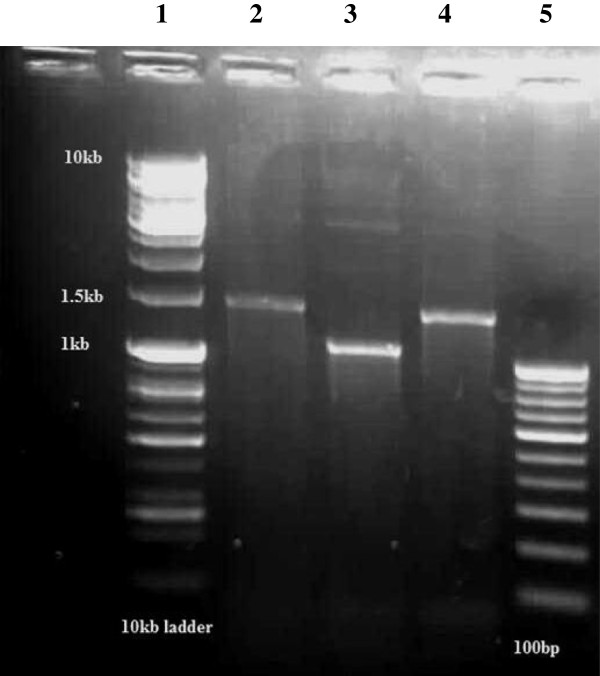
**Agarose gel electrophoresis of PCR assay of gene cassette amplification.** Lane 1, 10 kb ladder, Lane 2, *aadA6-orfD* gene cassette, Lane 3, *aadA13* Lane 4, *aadA6-orfD*, Lane 5, 100 bp ladder.

Using the hep58 and hep59 primers for gene cassette characterization, two different fragments sizes of approximately 1.6 kb and 1.2 kb were obtained for all the 31 *intI1*-positive isolates (Figure 2). Twenty-three (74%) isolates yielded a single fragment of approximately 1.6 kb while the remaining 8 isolates gave a single fragment of 1.2 kb. Sequence data [accession number JX195555] obtained from sequencing the 1.6 kb gene cassette fragment from strain ODM-24 gave 100% homology with *aadA6*, conferring resistance to streptomycin and spectinomycin and *orfD* of unknown function [25,26] whereas the 1.2 kb fragment of strain ODM-08 was 100% identical to *aadA13*, which also confer resistance to streptomycin and spectinomycin [accession number JX195556].

### Transfer of antibiotic resistance and genetic location of integrons

All the 31 class 1integron positive *P. aeruginosa* strains were tested for conjugal transfer of resistance plasmids and gene cassettes. Conjugation experiment was successful in 10 (32%) donor strains but none of the *E. coli* DHα transconjugants plasmids was positive for class 1 integron and gene cassette by PCR.

## Discussion

To our knowledge, this is the first study to report carriage of class 1 integrons and associated gene cassettes in *P. aeruginosa* isolates from Nigeria. The 57.4% incidence rate of class 1 integrons observed in this study is higher than previously reported rates of 41.5% from Brazil [3], 45.8% from China [13], and 56.3% from Iran [27]. The prevalence of class 1 integrons in clinical isolates of *P. aeruginosa* from Southwest Nigeria is of great concern because these genetic elements are highly stable among resistant pathogens; and also capable of easy spread and capture of other multidrug resistance gene cassettes leading to increase in resistance to broad-spectrum antibiotics [28]. Consistent with other previous studies that documented low detection of class 2 and class 3 integrons in clinical *P. aeruginosa*[13,29], none of the isolates in this study harboured class 2 or class 3 integron.

In Africa, class 1 integrons have been reported previously in clinical isolates of *P. aeruginosa.* Labuschagne *et al. *[30] reported *bla*_GES-5_ and *bla*_GES-5_-like genes as part of the variable region of class 1 integrons, occurring in three clinical *P. aeruginosa* isolates from South Africa. Another study reported class 1 integron containing *bla*_VIM-2_, *aacA7* and *aacA4*, as well as *aadB* and *arr6*, a novel rifampin resistance gene among 35 clonally related *P. aeruginosa* isolated from a hospital in Tunisia [31]. This suggests that class 1 integrons are strongly associated with multiple drug resistance and are frequently detected among clinical isolates of *P. aeruginosa *[32].

Clinically significant antimicrobial resistance was found among isolates that were positive for class 1 integrons, with notable high resistance rates to cefotaxime (77.4%), carbenicillin (80.6%), ceftriaxone (87.1%), streptomycin (90.3%) and tetracycline (100%). This is comparable with previously reported high rates of resistance to ceftriaxone (88.7%) and cefotaxime (90.1%) [5], tetracycline (100%) and gentamicin (78.6%) [29], among integron positive clinical *P. aeruginosa* from China. The high resistance rates observed in this study against beta-lactam antibiotics (ceftriaxone, cefotaxime, carbenicillin and piperacillin) is disturbing because resistance to this class of antibiotics in Gram-negative bacteria including *P. aeruginosa* is usually mediated by the production of extended-spectrum β-lactamase enzymes that are mostly plasmid encoded [33].

The gene cassettes mostly detected in all the class 1 integrons belong to *aadA* family conferring resistance to streptomycin and spectinomycin [25]. The *aadA6-orfD* gene cassette array derived from sequencing of the 1.6 kb gene cassette fragment from isolate ODM-24 showed complete homology with the *aadA6-ofrD* of class 1 integron reported from previous studies [25,34]. Naas *et al. *[25] first reported gene cassette *aadA6* as novel gene cassette in *P. aeruginosa*; and it has been subsequently reported to be highly conserved among the class 1 integrons gene cassettes of the Enterobacteriaceae most especially *E. coli *[35,36]. The complete sequence of *aadA13* obtained from sequencing ODM-08 was identical to previously reported *aadA13* sequences [accession numbers DQ779002, DQ779001]. Interestingly, there are few reports of *aadA13* in clinical strains of *P. aeruginosa*, unlike other *aadA* family, *aadA13* gene cassette are novel and rarely reported in *P. aeruginosa.* In a recent report, Yuan *et al.*[37] reported *aadA13* cassette, which was present in a new array of *aac(6′)-II-aadA13-cmlA8-oxa-10* gene cassette from *P. aeruginosa*.

*P. aeruginosa* is well known for harbouring multiple copies of plasmids, some of which are conjugative with resistance genes that are responsible for multiple drug resistance. *P. aeruginosa* strains from this study harboured 1 to 4 plasmids with sizes ranging from 2.2 kb to >58 kb. This findings contradict previous studies, [38,39] which reported low molecular weight plasmids (<2 kb) in clinical isolates of *P. aeruginosa* from Nigeria. The relationship between plasmid profiles and multiple drug resistance patterns observed in this study suggests that plasmids may have played a significant role in the multidrug resistance of *P. aeruginosa* strains from this study because multiple antibiotic resistance genes as well as virulence genes have often been found clustered together on a single plasmid [40].

Though, conjugation experiment was successful in 10 (32%) *P. aeruginosa* parental strains, PCR amplification with specific primers revealed the absence of integrons and gene cassettes among the transconjugants; indicating the non-transference of the integrons and associated gene cassettes along with the conjugative plasmids. This is probably suggestive of chromosomal location of the integrons and gene cassettes as previously observed in previous studies that non-plasmid lateral exchange of resistance regions may be common in *P. aeruginosa. *[32] In addition, our result also concurs with a recent paper on the dispersal of resistance regions from chromosomally located class 1 integrons possibly serving as the major genetic element of global dissemination in *P. aeruginosa*[41]*.*

In summary, our data demonstrate the presence of multiple drug resistant *P. aeruginosa* harbouring antibiotic resistant plasmids, class 1 integrons and gene cassettes that can be easily dispersed among other bacteria, resulting in the rapid spread of antibiotic resistance genes. Disturbingly, the widespread dissemination of the class 1 integron and associated gene cassettes in *P. aeruginosa* and other clinically important pathogens would gravely complicate treatments of infections in our hospitals if not properly monitored. Hence, functional surveillance of antimicrobial resistance and appropriate, effective measures geared towards curbing indiscriminate and unregulated use of antibiotics are urgently needed to prevent outbreaks of multidrug resistant bacteria in Southwest Nigeria hospitals.

## Competing interests

The authors declare no competing interests.

## Authors’ contributions

BTO and BAA planned this study. BTO performed the experiments under the guidance of RC. BTO wrote the manuscript. All authors read and approved the final manuscript.
